# Hill number as a bacterial diversity measure framework with high-throughput sequence data

**DOI:** 10.1038/srep38263

**Published:** 2016-11-30

**Authors:** Sanghoon Kang, Jorge L. M. Rodrigues, Justin P. Ng, Terry J. Gentry

**Affiliations:** 1 Department of Biology, Baylor University, Waco, TX, USA; 2 Department of Land, Air and Water Resources, University of California, Davis, Davis, CA, USA; 3 Department of Soil & Crop Sciences, Texas A&M University, College Station, TX, USA.

## Abstract

Bacterial diversity is an important parameter for measuring bacterial contributions to the global ecosystem. However, even the task of describing bacterial diversity is challenging due to biological and technological difficulties. One of the challenges in bacterial diversity estimation is the appropriate measure of rare taxa, but the uncertainty of the size of rare biosphere is yet to be experimentally determined. One approach is using the generalized diversity, Hill number (*N*_*a*_), to control the variability associated with rare taxa by differentially weighing them. Here, we investigated Hill number as a framework for microbial diversity measure using a taxa-accmulation curve (TAC) with soil bacterial community data from two distinct studies by 454 pyrosequencing. The reliable biodiversity estimation was obtained when an increase in Hill number arose as the coverage became stable in TACs for *a* ≥ 1. *In silico* analysis also indicated that a certain level of sampling depth was desirable for reliable biodiversity estimation. Thus, in order to attain bacterial diversity from second generation sequencing, Hill number can be a good diversity framework with given sequencing depth, that is, until technology is further advanced and able to overcome the under- and random-sampling issues of the current sequencing approaches.

Biodiversity has traditionally been considered to be a consequence of environmental processes, such as niche partitioning, resource distribution, and disturbances. In the last several decades, a new view of biodiversity as the predictor of environmental processes and functions gained interest^1^,^2^,^3^ and developed into the research field now regarded as biodiversity-ecosystem function (BEF)^4^,^5^,^6^. Bacteria have an intimately interactive relationship with its surrounding environment and ecosystem, and thus, bacetrial diversity has an important role in BEF research^7^,^8^. However, even determining a reasonable description of bacterial diversity is challenging due to the intrinsic properties of bacteria (e.g., debatable species concept, hyperdiversity, variable 16S rRNA gene copy number) and technological difficulties^9^,^10^,^11^,^12^. One of the challenges in bacterial diversity estimation is the capture of rare taxa (rare biosphere), which often occupy large portions of microbial diversity^13^,^14^,^15^; the experimental determination of the uncertainty involved is not yet available. Since 2005, the second generation sequencing technologies drastically advanced the capacity and the depth of microbial community sampling by sequencing. However, there is still bias associated with the experimental procedures, and sampling by sequencing is also known to be a less-than-complete representation^16^. Thus, reproducible estimation of biodiversity is not yet available^17^. One way to overcome this problem is to use statistical and mathematical biodiversity estimations^18^. However, most mathematical and statistical approaches of biodiversity estimation were developed for investigating less diverse organisms (e.g., plants and animals), which imposes an inheritant challenge in applying these tools to the analysis of bacterial communities due to their hyperdiversity. Therefore, a framework accomodating those challenges is needed for a reasonable bacterial diversity estimation using current available experimental resources.

Hill number (*N*_*a*_)^19^ was proposed as a unified diversity concept by defining biodiversity as a reciprocal mean proportional abundance and differently weighing taxa based on their abundances as follows:


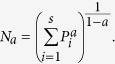


Parameter *a* determines special cases of Hill number, for example, *N*_*0*_ as number of taxa, *N*_*1*_ as exponential Shannon index, and *N*_*2*_ as reciprocal Simpson index^19^. Because of the generality and flexibility in controlling the effects of rare taxa in biodiversity measure, Hill number may be an excellent framework for bacterial diversity studies^9^. Recently, Haegeman *et al*.^20^ showed that the uncertainty associated with Hill numbers quickly increased to an uncontrollable range when *a* < 1 from the series of sequence data sets.

The consensus in bacterial diversity studies is that a fully exhaustive census may require an extremely large amount of resources for most natural ecosystems^11^,^21^. We argue that the “unsaturation” or asymptotic result in those rarefaction curves is due to the vast size of rare biosphere; thus, the saturated bacterial diversity may be obtainable with reasonable sequencing efforts using diversity measure framework of Hill number with differential weight on rare taxa. The goal of this study is to investigate the use of Hill number as a framework for reliable diversity estimation given sequencing depth.

## Results and Discussion

**Figure 1:**
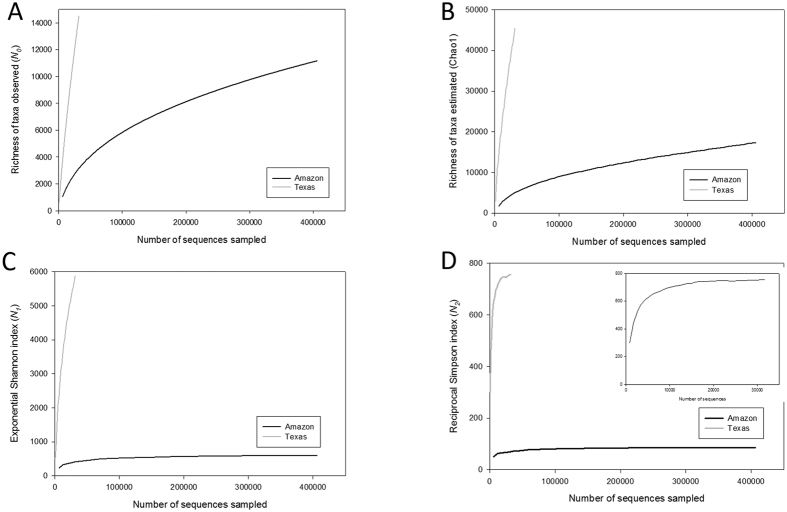
Smoothed taxa-accumulation curves (TACs) with different Hill numbers (**A** *N*_*0*_, **C** *N*_*1*_ and **D** *N*_*2*_) and Chao1 index (**B**) for both Amazon (66 samples) and Texas mine (36 samples) studies together. Insert is the Texas mine rarefaction curve, shown alone in order to better represent the trend due to the large difference in sequence reads between two data sets. Taxa (*N*_*0*_) represents unique OTU at 97% similarity cutoff.

The taxa-accumulation curves of the Amazon and Texas mine studies (Fig. 1, S1 and S2) show both similarities and differences in their patterns. The richness measures (*N*_*0*_ and Chao1 index) are far from saturated in both studies, and as the parameter *a* increased, the degree of diversity coverage increased, as well. The degree of coverage, however, was much less in the Texas mine study; only *N*_*2*_ was able to provide enough coverage (asymptote). Apparently, the difference is due to the depth of sampling (sequencing), which will be further discussed below with *in silico* analysis. The higher *a* represents increased insensitivity to the contributions by rare taxa to the overall biodiversity (γ diversity) and more robustness in doing so with reduced uncertainty^20^.

**Figure 2:**
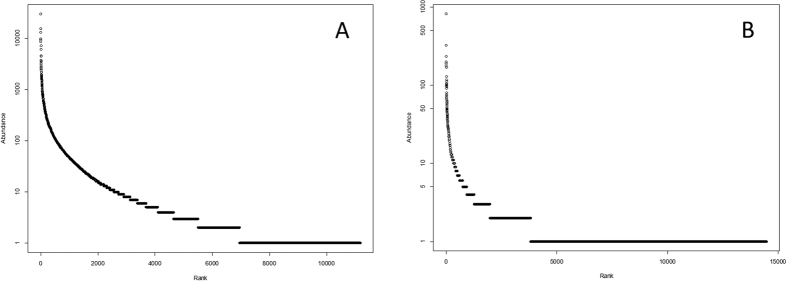
Rank abundance distribution plots (Whittaker plots) for Amazon (**A**) and Texas mine (**B**) studies. The best fit taxa abundance distribution (TAD) models are a log normal distribution for Amazon and a Zipf distribution for Texas mine data.

This analysis revealed an interesting pattern between the soil bacterial communities measured in very different sequencing depths from two distinct ecosystems. The observed taxa richness (*N*_*0*_) is fairly similar, but the difference becomes greater as *a* increases (Table S1) in that the Texas mine soil bacterial community is much more diverse than that of the Amazon soil samples. This is at least partly due to the abundant rare taxa, which should have caused rather low sampling completedness in Texas mine (~32%) compared to the Amazon samples (~65%)^22^. In the case of the Chao1 index, large numbers of singleton and doubleton in the Texas mine samples inflate the Chao1 index which is defined, in part, as the ratio between the square of the singleton frequency (*F*_1_), and times two of the doubleton frequency (*F*_2_) (Fig. 2B). It is impossible to determine how much of those singletons and doubletones are a part of real rare taxa and sequencing artifacts. However, because of the uncertainty, Hill number may be useful by enabling controlling of the contributions of rare taxa on determining diversity. Significant deviation (*D* = 0.17, *P* < 0.001) from a log-normal model also indicates incomplete sampling in the Texas mine microbial communities^23^. The large difference in the proportion of rare taxa between the two data sets also resulted in distinctive taxa abundance patterns (Fig. 2 and S3). Since the Texas mine samples were from the chronosequence of reclamation, the Zipf model is conceptually fitting^24^. However, under-sampling of the Texas data set may be contributing to the distinctive taxa abundance patterns, as well. To test the relationship between sampling degree and biodiversity coverage in TAC, we used randomly subsampled Amazon data between 25,000 and 400,000 reads in varing degrees (Fig. S4). Sufficient biodiversity coverage using TAC seems to be obtained with ~200,000 reads resulting in reliable biodiversity measures (*N*_*1*_ and *N*_*2*_).

The two data sets used here were suitable because they were prepared using almost identical procedures, but the sequencing depths were vastly different. A recent study using a mock community concluded that microbial composition results are influenced by the primers and sequencing platforms used^25^; thus, the compatible experimental procedure increases the credibility of the results. The diverse sequencing coverage is also useful because it could show the scale-independency of the analyses and results.

In conclusion, the hyperdiverse nature of microbiota in most ecosystems often results in random- and under-sampling, thus hampering reliable diversity estimations even with the technological advancementes made by the second generation sequencing technologies. Until a series of significant technological advancements in sampling coverage is available, the Hill number and TAC approach may be a suitable framework for reliable estimation of diversity and further applications in research studies like BEF and dimensions of biodiversity.

## Methods

We used a smoothed taxa-accumulation curve (TAC), which is often mis-labeled as a rarefaction curve, to investigate a reliable approach to estimate bacterial diversity from two 454 pyrosequence data sets. One data set is from soil samples in a chronosequence of reclaimed surface mine sites in East Texas (Texas study) and the other is from soil samples from an Amazonian rainforest that was converted to agricultural fields (Amazon study). Both data were prepared by very similar experimental and analytical procedures. Briefly, both studies used a PowerSoil DNA Isolation kit for DNA extraction (MoBio Laboratories) following manufacturer’s instruction and 454 GS FLX Sequencer (454 Life Sciences) for 16S rRNA gene sequencing at V4-V5 region (~350 bp). The quality processed sequences were analyzed using mothur software (v. 1.23.1)^26^ with SILVA and ribisomal database project (RDP) database for alignment and classification.

The depth of sequencing was quite different between the two studies: ~31,000 reads in the Texas mine sample in comparing mine reclaiming techniques (crosspit spreader, CP and mixed overburden, MO) and ~400,000 reads in the Amazon sample between forest and converted pasture. First, unique taxa (OTU_0.97_) richness (*N*_*0*_), Chao1 index^27^, exponential Shannon index (*N*_*1*_), and reciprocal Simpson index (*N*_*2*_) were calculated then used in TAC construction and by using EstimateS 9.1^28^ and R 3.1.3^29^. Rank abundance distribution (RAD) plots were prepared using vegan (2.2-1) and sads packages (0.2.4).

## Additional Information

**Accession codes:** Sequence data used for this study is available from NCBI Sequence Read Archive (SRA) under accession number SRP026369 (Texas Mine data) and FigShare, http://dx. doi.org/10.6084/m9.figshare.1547935 (Amazon data). 

**How to cite this article**: Kang, S. *et al*. Hill number as a bacterial diversity measure framework with high-throughput sequence data. *Sci. Rep.* **6**, 38263; doi: 10.1038/srep38263 (2016).

**Publisher’s note:** Springer Nature remains neutral with regard to jurisdictional claims in published maps and institutional affiliations.

## Supplementary Material

Supplementary Information

## References

[b1] Naeem S. . Biodiversity and ecosystem functioning: Maintaining natural life support processes (Ecological Society of America, Washington DC, 1999).

[b2] Naeem S., Thomson L. J., Lawlor S. P., Lawton J. H. & Woodfin R. M. Declining biodiversity can alter the performance of ecosystems. Nature 368, 734–737 (1994).

[b3] Tilman D. Biodiversity: population versus ecosystem estability. Ecology 77, 350–363 (1996).

[b4] Hector A. & Bagchi R. Biodiversity and ecosystem multifunctionality. Nature 448, 188–190, doi: 10.1038/nature05947 (2007). 17625564

[b5] Loreau M. . Biodiversity and ecosystem functioning: current knowledge and future challenges. Science 294, 804–808, doi: 10.1126/science.1064088 (2001). 11679658

[b6] Radchuk V., De Laender F., Van den Brink P. J. & Grimm V. Biodiversity and ecosystem functioning decoupled: invariant ecosystem functioning despite non-random reductions in consumer diversity. Oikos 125, 424–433, doi: 10.1111/oik.02220 (2016).

[b7] Philippot L. . Loss in microbial diversity affects nitrogen cycling in soil. ISME J. 7, 1609–1619, doi: 10.1038/ismej.2013.34 (2013). 23466702 PMC3721106

[b8] van der Heijden M. G. A., Bardgett R. D. & van Straalen N. M. The unseen majority: soil microbes as drivers of plant diversity and productivity in terrestrial ecosystems. Ecol. Lett. 11, 296–310, doi: 10.1111/j.1461-0248.2007.01139.x (2008). 18047587

[b9] Bent S. J. & Forney L. J. The tragedy of the uncommon: understanding limitations in the analysis of microbial diversity. ISME J. 2 (2008). 10.1038/ismej.2008.44 18463690

[b10] Escalas A. . A unifying quantitative framework for exploring the multiple facets of microbial biodiversity across diverse scales. Environ. Microbiol. 15, 2642–2657, doi: 10.1111/1462-2920.12156 (2013). 23731353

[b11] Roesch L. F. W. . Pyrosequencing enumerates and contrasts soil microbial diversity. ISME J. 1, 283–290 (2007). 18043639 10.1038/ismej.2007.53 PMC2970868

[b12] Větrovský T. & Baldrian P. The Variability of the 16S rRNA Gene in Bacterial Genomes and Its Consequences for Bacterial Community Analyses. PLOS One 8, e57923, doi: 10.1371/journal.pone.0057923 (2013). 23460914 PMC3583900

[b13] Boeken B. & Shachak M. Linking community and ecosystem processes: The role of minor species. Ecosystems 9, 119–127 (2006).

[b14] Sogin M. L. . Microbial diversity in the deep sea and the underexplored “rare biosphere”. Proc. Natl. Acad. Sci. USA 103, 12115–12120 (2006). 16880384 10.1073/pnas.0605127103 PMC1524930

[b15] Lynch M. D. J. & Neufeld J. D. Ecology and exploration of the rare biosphere. Nat Rev Micro 13, 217–229, doi: 10.1038/nrmicro3400 (2015). 25730701

[b16] Zhou J. . Random Sampling Process Leads to Overestimation of β-Diversity of Microbial Communities. mBio 4, doi: 10.1128/mBio.00324-13 (2013). PMC3684833 23760464

[b17] Zhan A. . Reproducibility of pyrosequencing data for biodiversity assessment in complex communities. Methods in Ecology and Evolution 5, 881–890, doi: 10.1111/2041-210X.12230 (2014).

[b18] Hughes J. B., Hellmann J. J., Ricketts T. H. & Bohannan B. J. M. Counting the uncountable: statistical approaches to estimating microbial diversity. Appl. Environ. Microbiol. 67, 4399–4406 (2001). 11571135 10.1128/AEM.67.10.4399-4406.2001 PMC93182

[b19] Hill M. O. Diversity and evenness: a unifying notation and its consequences. Ecology 54, 427–432 (1973).

[b20] Haegeman B. . Robust estimation of microbial diversity in theory and in practice. ISME J. 7, 1092–1101, doi: 10.1038/ismej.2013.10 (2013). 23407313 PMC3660670

[b21] Quince C., Curtis T. P. & Sloan W. T. The rational exploration of microbial diversity. ISME J. 2, 997–1006 (2008). 18650928 10.1038/ismej.2008.69

[b22] Coddington J. A., Agnarsson I., Miller J. A., Kuntner M. & Hormiga G. Undersampling bias: the null hypothesis for singleton species in tropical arthropod surveys. J. Anim. Ecol. 78, 573–584 (2009). 19245379 10.1111/j.1365-2656.2009.01525.x

[b23] Ulrich W., Ollik M. & Ugland K. I. A meta-analysis of species-abundance distributions. Oikos 119, 1149–1155 (2010).

[b24] Wilson J. B. Methods for fitting dominance/diversity curves. J. Veg. Sci. 2, 35–46 (1991).

[b25] Fouhy F., Clooney A. G., Stanton C., Claesson M. J. & Cotter P. D. 16S rRNA gene sequencing of mock microbial populations- impact of DNA extraction method, primer choice and sequencing platform. BMC Microbiol. 16, 1–13, doi: 10.1186/s12866-016-0738-z (2016). 27342980 PMC4921037

[b26] Schloss P. D. . Introducing mothur: Open-source, platform-independent, community-supported software for describing and comparing microbial communities. Appl. Environ. Microbiol. 75, 7537–7541 (2009). 19801464 10.1128/AEM.01541-09 PMC2786419

[b27] Chao A. Nonparametric estimation of the number of classes in a population. Scand J Statist 11, 265–270 (1984).

[b28] EstimateS: Statistical estimation of species richness and shared species from samples. Version 9 (2013).

[b29] R: A language and environment for statistical computing. (R Foundation for Statistical Computing, Vienna, Austria, 2015).

