# Supplementary material for: Hill number as a bacterial diversity measure framework with high-throughput sequence data
Source: Sci Rep. 2016 Nov 30;6:38263. doi: 10.1038/srep38263 (PMC5128788; doi:10.1038/srep38263)

Supporting Information

Hill number is a reliable bacterial diversity measure with high-throughput sequence data

Sanghoon Kang[1*], Jorge L.M. Rodrigues[2], Justin P. Ng[3] & Terry J. Gentry[3]

1. Department of Biology, Baylor University, Waco, TX, USA
2. Department of Land, Air and Water Resources, University of California, Davis, Davis, CA, USA
3. Department of Soil & Crop Sciences, Texas A&M University, College Station, TX

Corresponding author:
Sanghoon Kang
Department of Biology, Baylor University
One Bear Place #97388
Waco, TX 76798 USA
Phone:1-254-710-2140
Fax:1-254-710-2969
sanghoon_kang@baylor.edu

Figure S1. TACs with different Hill numbers (A. N_0, C. N_1, D. N_2) and Chao1 index (B) for Amazon samples between forest and converted pasture conversion (33 samples each)

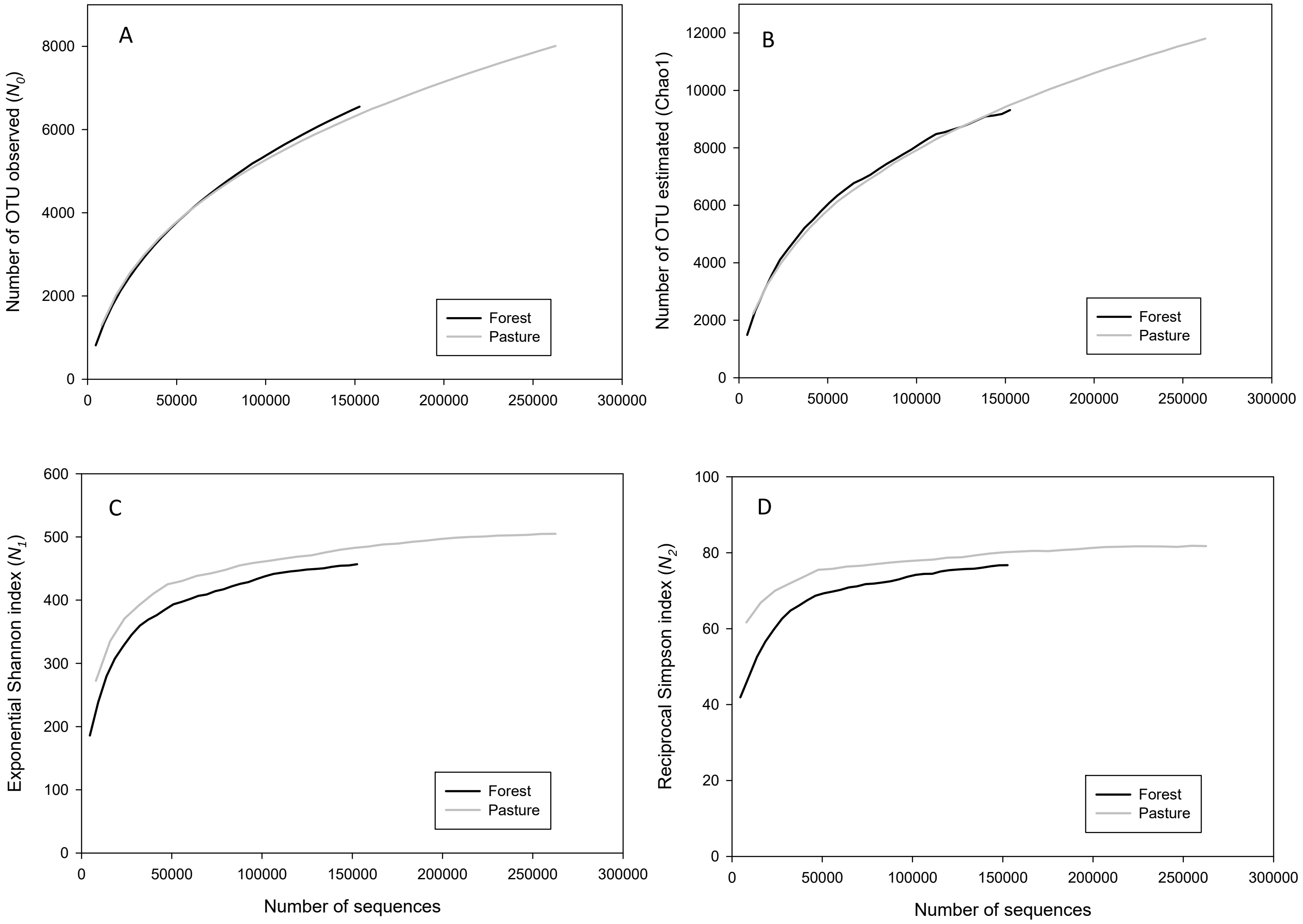

Figure S2. TACs with different Hill numbers (A. N_0, C. N_1, D. N_2) and Chao1 index (B) for reclaimed surface mine samples between two reclamation techniques used (20 samples for crosspit spreader (CP) and 12 samples for mixed overburden (MO)). Observed OTU was extrapolated beyond 12th samples for MO using approach by Colwell *et al.* (26).

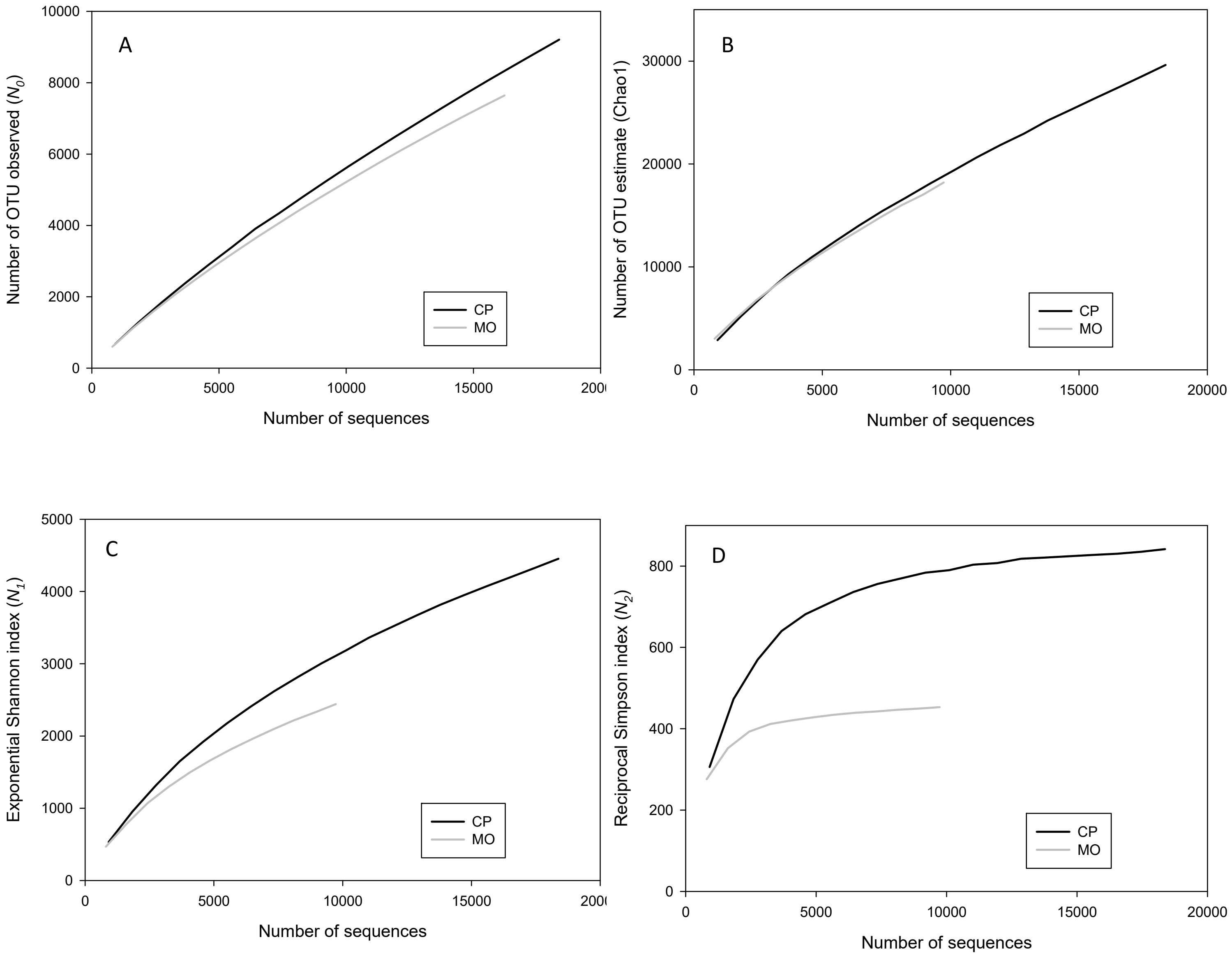

Table S1. Estimated microbial biodiversity by different biodiversity measures for both Amazon and Texas mine studies.

		Amazon (66 samples)			Texas mine (36 samples)		
		Total	Forest	Pasture	Total	CP	MO
N_0		11174	6550	8009	14492	9200	7641.56[b]
	CI	135.4	96.2	107.7	201.8	162.3	190
Chao1		17291.27	9313.34	11800.63	45489.55	20650.45[c]	18198.36
	CI[a]	400.8	226.7	305.1	1222.3	870.9	1355.8
N_1		592.85	456.71	505.01	5871.8	3360.52[c]	2440.86
N_2		85.91	76.7	81.73	755.27	803.44[c]	452.93

[a]95% confidence interval. CI of Chao1 is the average between upper and lower CI bound.
[b]observed taxa richness was extrapolated beyond 12th sample using approach by Colwell et al 2012.
[c]Chao1 index, exponential Shannon index (N_1) and reciprocal Simpson index (N_2) were rarefied at 12th sample.

Figure S3. Frequency distribution plot (Preston plot) of Amazon (A) and Texas mine study (B).Figure S4.

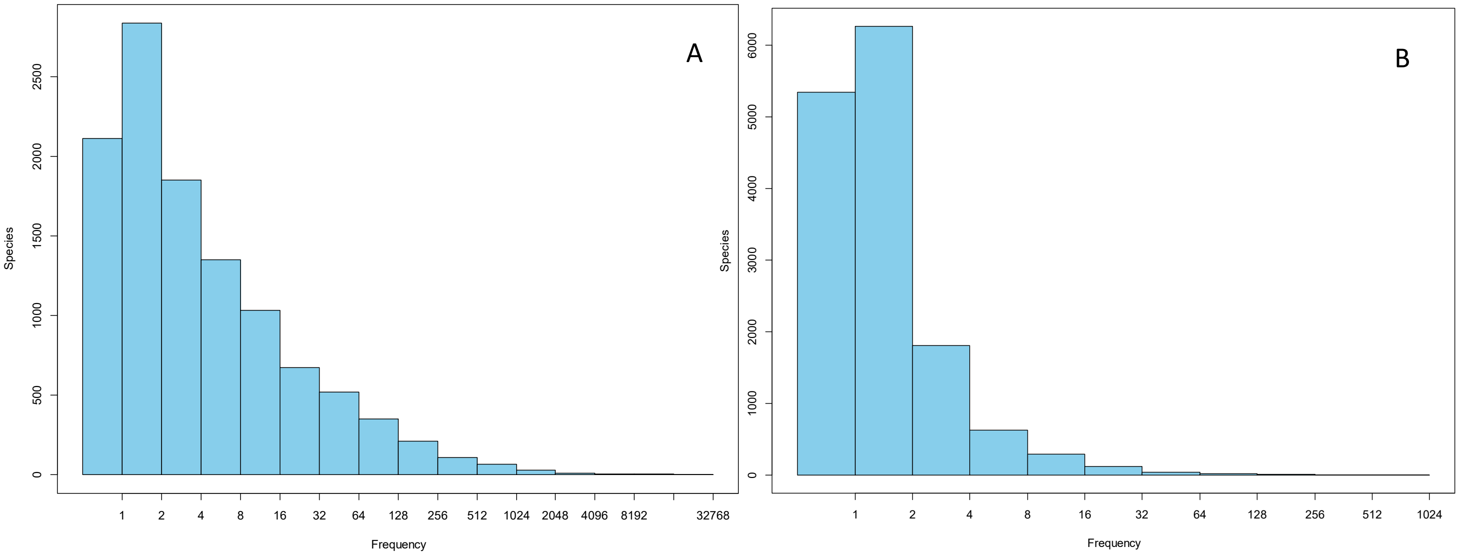

Figure S4. TACs with reliable biodiversity measure (A. N_1, B. N_2) with subsampled Amazon study data set.

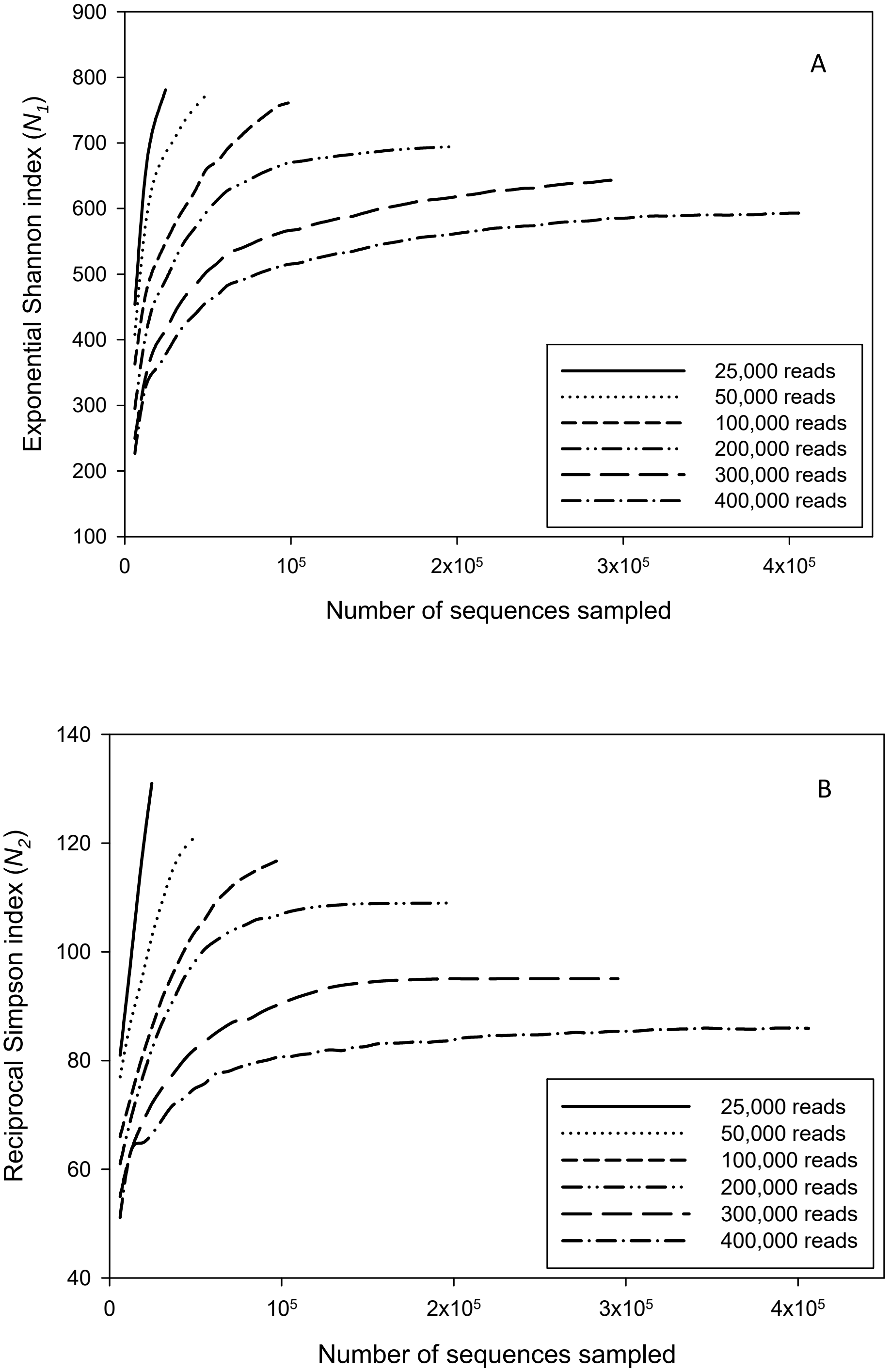

Supplement: Supplementary Information [file srep38263-s1.pdf]
